# Plant Cell Wall Proteomics: A Focus on Monocot Species, *Brachypodium distachyon*, *Saccharum* spp. and *Oryza sativa*

**DOI:** 10.3390/ijms20081975

**Published:** 2019-04-23

**Authors:** Maria Juliana Calderan-Rodrigues, Juliana Guimarães Fonseca, Fabrício Edgar de Moraes, Laís Vaz Setem, Amanda Carmanhanis Begossi, Carlos Alberto Labate

**Affiliations:** Department of Genetics, Max Feffer Laboratory of Plant Genetics, “Luiz de Queiroz” College of Agriculture, University of São Paulo, CP 83, 13400-970 Piracicaba, SP, Brazil; j.g.fonseca@usp.br (J.G.F.); fabricioedgar.m@gmail.com (F.E.d.M.); lala_arnz@hotmail.com (L.V.S.); amanda.lpp@hotmail.com (A.C.B.); calabate@usp.br (C.A.L.)

**Keywords:** plant cell wall, proteome, monocot, stiff brome, rice, sugarcane, *Brachypodium distachyon*, *Saccharum* spp., *Oryza sativa*

## Abstract

Plant cell walls mostly comprise polysaccharides and proteins. The composition of monocots’ primary cell walls differs from that of dicots walls with respect to the type of hemicelluloses, the reduction of pectin abundance and the presence of aromatic molecules. Cell wall proteins (CWPs) differ among plant species, and their distribution within functional classes varies according to cell types, organs, developmental stages and/or environmental conditions. In this review, we go deeper into the findings of cell wall proteomics in monocot species and make a comparative analysis of the CWPs identified, considering their predicted functions, the organs analyzed, the plant developmental stage and their possible use as targets for biofuel production. *Arabidopsis thaliana* CWPs were considered as a reference to allow comparisons among different monocots, i.e., *Brachypodium distachyon*, *Saccharum* spp. and *Oryza sativa*. Altogether, 1159 CWPs have been acknowledged, and specificities and similarities are discussed. In particular, a search for *A. thaliana* homologs of CWPs identified so far in monocots allows the definition of monocot CWPs characteristics. Finally, the analysis of monocot CWPs appears to be a powerful tool for identifying candidate proteins of interest for tailoring cell walls to increase biomass yield of transformation for second-generation biofuels production.

## 1. Introduction

The plant cell wall confines the cell volume and serves as protection against stresses, being responsible for the plant shape, enabling trees to be several meters high. In addition to these functions, the cell wall is the most external part of the cell, and as such, interacts with the apoplast, which is also essential in virtually all cell processes, including division, expansion, differentiation [1], growth and signaling [2]. This versatility implies that the mechanisms involved in a great deal of the cell wall functions have not been completely depicted.

The plant cell wall is a dynamic structure that undergoes changes during development [3]. The cell wall is mainly composed of wall polysaccharides, such as cellulose, hemicellulose, pectin, and proteins [4]. In some cell types, lignin and other compounds might be found, as well. Carbohydrates account for around 90% of the cell wall mass, and proteins around 10% for dicots [5] and 1% for monocots [6]. Cell wall proteins (CWPs) are understood to be proteins directed towards the secretory pathway, such as structural proteins linked to the wall and those secreted into the apoplast and extracellularly [7].

Some characteristics are commonly found in classical CWPs: an N-terminal cleavage signal-peptide, responsible for guiding these proteins to the endoplasmic reticulum (ER) [8]; lack of the ER-retention C-terminal motif KDEL/HDEL, which avoids the secretion of proteins [9,10]; and the absence of a transmembrane domain that retains proteins in the plasma membrane [11]. The secretory pathway directs proteins from ER to the Golgi apparatus, where they are packed into vesicles and guided to the cell wall and the extracellular matrix. 

CWPs encompass hundreds of molecules presenting distinctive roles and are related to the modification of the cell wall components, signaling, interaction with the apoplast and the plasma membrane, and with virtually all the cell wall-related processes [12,13]. CWPs are usually divided into categories with respect to their functionality, such as proteins acting on carbohydrates, oxido-reductases, proteins related to lipid metabolism, and proteases, which represent the most abundant classes [7]. 

Besides being divided regarding their functional roles, CWPs can also be classified according to their type of link to the cell wall, such as structural proteins, which are strongly bound to the wall components, and loosely and weakly wall-bound proteins [13]. Loosely bound proteins are not linked to the cell wall polysaccharides and are able to move unconnected in the intercellular space. Weakly bound proteins interact with the matrix by Van der Waals, hydrogen bonds or ionic forces, and are related to remodeling, signaling, interactions with the plasma membrane and defense. Strongly bound proteins can be connected by covalent bonds [14]. 

To increase the comprehension of cell wall-related processes, several reports have characterized plant CWPs. To accomplish this task, specific methods of CWP extraction and data analysis had to be established, since these proteins are usually lost or in a low abundance in total proteins extraction surveys [15]. The constitution and structure of the CWPs collection are diverse due to the developmental stage, organ, tissue and species of the analyzed material; thus, it is possible to make a comparison among these different treatments and relate them physiologically [7]. Initial studies have been made on *Arabidopsis thaliana* [11,16,17,18,19,20,21,22]. Since then, CWPs from several other species have been identified by mass spectrometry. In *A. thaliana*, about one half of the predicted existing CWPs were identified, being the most studied plant at present, with 935 proteins already identified [11,16,17,18,19,20,21,22]. In addition to providing an overview of proteins present in a biological context, the identification of CWPs in *A. thaliana* enabled the selection of candidates to have their structure and function further investigated.

Over the course of time, several other studies have been performed with model plants and crops: Brassica [23,24], *Zea mays* [25], *Cicer arietinum* [26], *Vitis vinifera* [27], *Oriza sativa* (rice) [28,29,30,31], *Nicotiana tabacum* [32], *Brachypodium distachyon* (stiff brome) [33,34,35,36], *Saccharum* spp. (sugarcane) [37,38,39,40], among others. Due to this functional diversity, CWPs data present differences between monocots and dicots related to the cell wall structure and composition [41], probably due to both metabolisms’ specificities. As other C3-dicots, *A. thaliana* has type I walls [42], whereas grasses, such as *Saccharum* spp., *B. distachyon*, *Sorghum bicolor* and *Setaria viridis*, present C4 metabolism and type II cell walls. *O. sativa* is a C3-monocot that also possesses type II walls [42]. Characteristic differences can be predicted when comparing CWPs collection from dicots and monocots, as their cell wall structures and components are diverse. For instance, the cell wall proteome of grasses generally consists of a higher proportion of oxido-reductases, and it has been suggested that this is due to the presence of aromatic compounds in the type II primary cell walls [33]. In summary, type II walls are characterized by a low proportion of pectins and xyloglucans, and high content of glucuronoarabinoxylans and mixed linked β-d-glucan, resulting in absent woody stems and branches [42,43], in comparison to dicots. Type II cell walls also contain ferulic and coumaric acids and more complex arabinoxylans in secondary walls. Moreover, the arabinosyl side chains inside arabinoxylans may be cross-linked with lignin through feruloyl esters [6,42]. One hypothesis for the selective advantage for the appearance of type II wall plants is that they are not as high and as reinforced as, for example, tall trees, so they focus their energy on rapid growth and more efficient reproductive strategies directed to occupy habitats that are not very suitable for trees [43]. 

Many of the monocots have C4 metabolism, presenting a higher photosynthetic efficiency, making them commercially valuable plants. As an example, *Saccharum* spp. has been used from more than three decades ago up to the present day as a raw material for the production of food, energy and co-products in Brazil [44]. Moreover, the study of CWPs in crops presents extra challenges compared to model plants, as less genetic and molecular data is usually available.

There is a need for more information about cell wall structure, components and their roles. The identification of CWPs allows new targets for further investigation and elucidation of pathways in order to better understand cell wall functions. Knowing that more than 10% of the plant’s genome is related to the cell wall biogenesis [45], and as there are fewer studies on CWPs in monocots than dicots, it is important to gather and analyze CWP data from monocots, which would provide new insights about their unique metabolism and the specificities of their cell walls. 

In this review, we compiled the information on monocots cell wall proteomics till date, except for studies using special treatments or stress-related data. Thus, *B. distachyon*, *Saccharum* spp. and *O. sativa* were chosen for this review. For each species, we searched for the *A. thaliana* sequences with the highest identities (BLASTp) in order to enable a comparison among all of them. Similar and identical *A. thaliana* sequences were found in monocots, and similarity in functional classes could also be established, as well as some specificities for each species. In this review, when it is mentioned that one *A. thaliana* CWPs was identified in the repertoire of the monocots, it means that the monocot CWP sequence matched that *A. thaliana* protein after BLASTp analysis.

## 2. Methods of Monocots CWPs Extraction and Analysis

Different methods of extraction of CWPs have been developed over the years. One of the biggest challenges is to isolate the cell wall with minimum contamination by intracellular or membrane proteins. Thus, the subcellular fractionation before proteomic analysis can be a useful strategy for acquiring a representative extract of CWPs and reducing contamination by proteins from other organelles. Protocols involving tissue grinding and centrifugation to generate a density gradient enable the separation of fractions that are highly enriched with specific cell compartments. However, such methods will also lead to plasma membrane disruption, which may result in subsequent contamination of cell wall subfraction. On the other hand, methodologies that do not break the cell structure can be less efficient, as the extraction buffers need to come into contact with the CWPs in order to extract them [17]. Thereby, methods of CWPs extraction can be divided into destructive and non-destructive techniques. 

Regarding CWP extraction, the situation is not just one-method-fits-all, but rather the species, the organ and the targeted subset of proteins have to be considered in order to choose the most adequate protocol(s). The destructive techniques used for monocot CWP extraction utilize tissue grinding followed by a growing gradient of sucrose with a low ionic strength buffer to allow the sedimentation and isolation of the cell wall while preserving the ionic bonds. This gradient is able to eliminate organelles and other molecules less dense than the cell wall polysaccharides. Then, the last step is dedicated to washing away intracellular proteins that have remained trapped in the cell wall matrix through extensive washing on a polymer net. After cell wall isolation, the destructive protein extraction usually relies on salts such as calcium and lithium chloride to successfully liberate the wall-bound proteins [46]. Calcium chloride (CaCl_2_) has the ability to collect CWPs because acidic and neutral carbohydrates strongly chelate calcium, and thus proteins weakly bound to the walls’ polysaccharides can be solubilized by CaCl_2_ through a competition mechanism [47]. In addition, lithium chloride (LiCl) is used to extract mostly hydroxyproline-rich glycoproteins [17]. The non-destructive CWPs extraction technique used for the monocots here revised is based on vacuum infiltration of the plant samples with solutions containing the same salts used for the destructive method mentioned above, followed by centrifugation of these samples to release the extracted CWPs [17].

Regarding mass spectrometry (MS), different strategies have been used for the study of monocot CWPs. These include previous separation by 1D-polyacrylamide gel electrophoresis (1D-PAGE) prior to tryptic digestion or shotgun analysis by liquid chromatography-tandem mass spectrometry (LC-MS/MS). Following the identification of proteins through MS and bioinformatics, data analysis is essential for subcellular and functional predictions. Table 1 summarizes the plant samples, isolation of CWPs and MS techniques used in monocot CWP studies.

*B. distachyon* is a model species for temperate grasses with a fully sequenced genome, in addition to being easily grown and closely related to biomass crops. In this species, nine different plant samples have been analyzed, including young and mature leaves, apical and basal internodes, seeds and seedlings at different developmental stages [33,34,35,36]. These studies could point to specificities in different organs and developmental stages analyzed, especially in glycoside hydrolases and oxido-reductases. Fewer proteins were extracted from mature organs in comparison to young ones, which was attributed to a higher level of cell wall polymers cross-linking [33]. *Saccharum* spp. is a crop of special economic interest, as it is one of the major sources of sugar and bioethanol. For this crop, ten different plant samples were surveyed, including suspension cells culture, young and mature leaves, and basal and apical internodes at different developmental stages [37,38,39,40,48]. *O. sativa* was one of the first plant species with a sequenced genome and it is an economically relevant crop worldwide. *O. sativa* CWP surveys used five plant samples, such as suspension cells culture, culture media, roots and leaves [28,29,30,31]. In the next sections, when a CWP is mentioned as identified or not in monocots or *A. thaliana*, it means that this protein was or was not identified by MS in the mentioned CWP studies, respectively. When discussing the gene sequences corresponding to these CWPs or functional studies on them, this information is specified in the text and referenced properly.

## 3. Functional Class Distribution in Monocots

**Figure 1:**
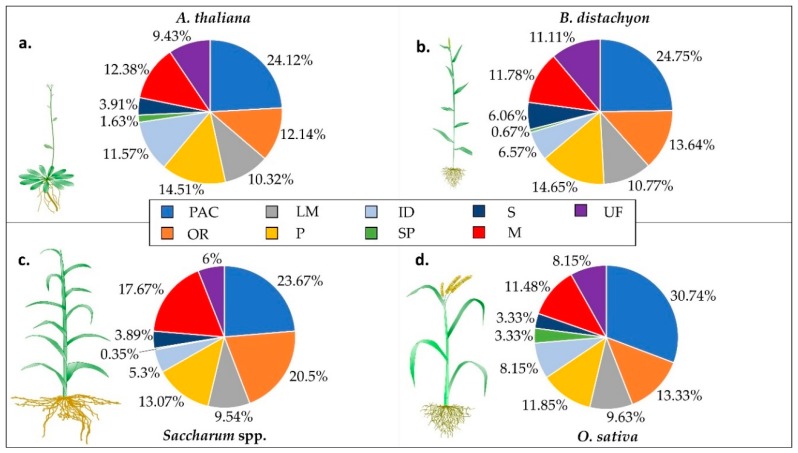
Percentage of CWP functional classes in the species *A. thaliana* (**a**), *B. distachyon* (**b**), *Saccharum* spp. (**c**) and *O. sativa* (**d**). Plant drawings are not to scale. Functional class abbreviations: proteins acting on carbohydrates (PACs), oxidoreductases (ORs), proteases (Ps), proteins related to lipid metabolism (LMs), proteins possibly involved in signaling (Ss), proteins with predicted interaction domains (IDs), miscellaneous proteins (Ms), proteins of unknown function (UFs) and structural proteins (SPs).

The number of non-redundant proteins was collected from each species and sorted into functional classes [28,29,30,31,33,34,35,36,37,38,39,40,48]. Although the results are not equally comparable, since different organs and several methods of CWP extraction and MS analysis were used, we were able to retrieve information regarding the functional classes and unique proteins in each species. Currently, the highest number of identified CWPs in monocots is from *B. distachyon*, comprising 594 proteins. *Saccharum* spp. is the second, with 283, and then *O. sativa*, with 270 identified CWPs. Altogether, 1159 CWPS proteins were identified, corresponding to 466 *A. thaliana* sequences. These proteins were divided into nine functional classes, according to Jamet et al. [7]: proteins acting on carbohydrates (PACs), oxidoreductases (ORs), proteases (Ps), proteins related to lipid metabolism (LMs), proteins possibly involved in signaling (Ss), proteins with predicted interaction domains (IDs), miscellaneous proteins (Ms), proteins of unknown function (UFs) and structural proteins (SPs). The functional classification of the CWPs from the monocots can be seen in Figure 1. 

To allow some comparison among the different CWPs on monocots, we performed BLASTP with *A. thaliana* protein sequences against *B. distachyon*, *O. sativa*, and *Saccharum* spp. sequences (using default parameters) and selected the first-ranked *A. thaliana* sequence. These monocot species were selected since they have a comparable number of CWPs already identified by mass spectrometry with no particular treatment. WallProtDB [48] was the database used to retrieve these data. Only proteins predicted to be secreted were considered. 

Taking the mentioned data together [28,29,30,31,33,34,35,36,37,38,39,40,48], as can be seen in Figure 1, the proportion of PACs inside the CWPs were similar among the three species, and ranged from around 20 (*Saccharum* spp.) to 30% (*O. sativa*), which is comparable to *A. thaliana* (~24%). For *O. sativa* and *B. distachyon*, the percentages of ORs were similar to *A. thaliana*—about 12 to 13%. Conversely, *Saccharum* spp. presented the highest proportion of ORs—20%. The LM percentages in all three species varied from around 9 to 11%, which was also similar to that of *A. thaliana*. Except for *B. distachyon*, the percentages of Ps in monocots were slightly lower than in *A. thaliana*. IDs were much lower in all the monocots in comparison with *A. thaliana*. In grasses, the percentage of SP was much lower than in *A. thaliana*. The percentages of Ms and UFs were higher and lower in *Saccharum* spp. in comparison with the other species, respectively.

### 3.1. Proteins Acting on Carbohydrates

Regarding PACs, from the 297 identified in cell wall proteomes, 111 are non-redundant *A. thaliana* sequences identified through BLASTp. Of these, 79 were also identified in *A. thaliana* CWP studies. Among the 297, several members of Glycosyl Hydrolase (GH) families 1 and 17 were identified. Other families, such as GH5, 13, 16, 32, 35 and 38, were also present. GHs are proteins involved in cell wall carbohydrates remodeling and can be regulated during development. These families are used in enzymatic cocktails for biomass degradation in second-generation ethanol production [49]. Literature data suggests that grasses present fewer GH1, GH16, GH28 and GH35 members than dicots and more GH5, GH13, GH18 and GH51 members [33,50], which is consistent with our comparative analysis on monocots *vs*. *A. thaliana*, in general. 

The GH1 AT1G61820 is a β-glucosidase named BGLU46, identified in *A. thaliana* and *B. distachyon* CWP studies. It has been suggested that coniferin is the substrate for BGLU46 and BGLU45, and that monolignol glucosides are a source of storage monolignols instead of direct precursors of lignin in Angiosperms. These storage monolignols could be metabolized under stress conditions, which would lead to lignin synthesis de novo [51].

The proportion of GH3 was slightly higher in monocots. Some *Saccharum* spp. GH3 have been found to be possible β-xylosidases, which catalyze the hydrolysis of xylose from xylo-oligosaccharides. In barley, a type II-wall C3, a binding site for (1 → 3, 1 → 4)-β-d-glucans was identified [52].

GH5 are more numerous in monocots (Os05g0244500, Os10g0370500, Bradi2g31690, Bradi5g13550 and Bradi5g13560), and this data may correlate with the fact that (1,3) (1,4)-β-d-glucans are their putative substrates [53]. *O. sativa* studies identified several GH13 (Os08g0473600, Os09g0457800, Os08g0473900, Os09g0457400, Os06g0713800, Os02g0765600 and Os09g0457600), all α-amylases that are capable of hydrolizing 1,4-α-glucosidic linkages [49], which is consistent with *O. sativa* endogenous metabolism. Starch is a plant carbohydrate often linked to storage organs, and its breakdown is mediated by amylases. In grains, there is an extracellular matrix enriched in starch, which is degraded by secreted enzymes, the alpha-amylases [4]. 

*Saccharum* spp. GH17, such as GH1, are probably β-glucosidases [49]. The proportion of GH17 was also higher in monocots (see Supplementary Table S1 for accession numbers). The presence of GH17 in monocots is not surprising, since, as mentioned, type II cell walls present mixed (1,3)(1,4)-β-d-glucans as the principal hemicellulose [54], which is the substrate for GH17 that displays glucan-1,3-β-glucosidase activity. These enzymes are used in enzymatic cocktails for biomass deconstruction and are considered one of the most efficient enzymes in breaking glycosidic bonds in hemicelluloses [55].

GH18 substrates are not currently known, but they could be xylanase inhibitors that promote cell wall extension or chitinases involved in cell signaling or pathogen response [56,57,58]. The proteins Bradi3g26840, Bradi3g26850, Os10g0416100 and SCQGRT3044B10 are all GH18 that did not match any *A. thaliana* sequence record. Interestingly, these *B. distachyon* GH18 (Bradi3g26840 and Bradi3g26850) were phylogenetically grouped into a separate clade in the GH18 family, and indeed, *A. thaliana* did not present any GH inside this clade [50]. Thus, because of their high specificity, the GH18 enzymes are excellent candidates for further investigation in order to unravel the structure of monocot cell walls. Additionally, as grasses are used as raw materials for biofuels production, the identification of GH functions could be valuable in solving bottlenecks related to biomass deconstruction.

*At*CWIN1 (AT3G13790) is a cell wall invertase from the GH32 family, and was identified in all CWP studies, except in *O. sativa*. *At*CWIN1 regulates carbon partitioning by cleaving apoplastic sucrose and helping in the process of carbon import into the cell, which is the role of membrane sugar transporters [59]. 

GH families 16 and 35 have more members in the *A. thaliana* proteome, and a possible explanation is that their substrates are xyloglucans and galactans, the last related to pectins. Galactan is involved in xyloglucan structure and mediates the interaction between xyloglucan and cellulose, in *A. thaliana*. Interestingly, the *At*BGAL1 (AT3G13750) from GH35 family, identified in *A. thaliana* and *B. distachyon*, act with BGAL3, identified in *A.thaliana* only, during cell elongation [60]. Whether this enzyme was poorly identified in monocots because of the low content of xyloglucans needs further investigation. The principal xyloglucan β-galactosidase in *A. thaliana*, *At*BGAL10, was also identified in all cell wall proteomes, with the exception of *O. sativa*.

More members of GH51 were found in *B. distachyon* (Bradi4g26270, Bradi1g63990, Bradi4g43710 and Bradi1g57017) than in *A. thaliana*. These GH51 are probably α-l-arabinofuranosidases [49], a type of enzyme used for monocots biomass deconstruction, as they are rich in arabinoxylans. α-l-arabinofuranosidases act on hydrolysis of α-l-arabinofuranoside in α-l-arabinosides, together with hemicellulases, resulting in hemicellulose hydrolysis [49]. It is important to mention that more studies related to substrate specificities and protein structure are necessary to establish the function of these proteins. 

In addition to cellulose and hemicellulose, pectin is one of the main constituents of the primary cell wall. Wall porosity, charge density and microfibril spacing are some of the functional roles of pectin [61]. Consistently with the fact that type II-wall plants have lower pectin content, Pectin Methyl Esterases (PMEs) and Pectate Lyase-like proteins were more represented in *A. thaliana*. After the transport from the Golgi apparatus to the cell wall, pectin is partially deesterified by PMEs, exposing a carboxyl group on galacturonosyl residues and allowing the pectin to be stiffened by ionic crossbonding with calcium ions [62]. The degree of methylation impacts on the wall stiffening and access to enzymes [63].

### 3.2. Oxidoreductases

ORs mostly comprise several class III peroxidases (Prxs), multicopper oxidases, plastocyanins, berberine-bridge enzymes (BBEs) and blue copper-binding proteins. Prxs, part of large multigenic families, can either oxidize phenolic compounds, and consume hydrogen peroxide or generate reactive oxygen species [64]. They have been involved in several functional roles, such as cell elongation, lignin metabolism, stress responses and germination (reviewed by [64]). As Prxs are versatile proteins, they can both promote cell wall expansion or the crosslinking of its components, favoring cell wall strengthening [65]; it is difficult to establish a correlation between their higher or lower proportion and their metabolic function. Among the ORs, monocots show a slightly higher percentage of Prxs, which may be related to the fact that Poaceae presents additional groups of paralogous Prxs genes in comparison to *A. thaliana* and other dicots [66]. Sugarcane commercial varieties are highly polyploid and aneuploid plants, usually resulting from the interspecific hybridization of *Saccharum officinarum* and *S. spontaneum* [67]. The higher proportion of ORs in *Saccharum* spp., mostly Prxs, compared to the other monocots may be due the high level of ploidy of this crop, but this observation is speculative. Different Prxs were identified when using destructive and non-destructive CWPs extraction in young *Saccharum* spp. culms [38], which was suggested to be due to a differential level of pectin-binding capacity, as Prx with a Ca^+2^-pectate binding domain would be more difficult to extract using the infiltration technique. Interestingly, *At*Prx34 (AT3G49120) and 36 (AT3G50990) were only identified in the *A. thaliana* cell wall proteome, which is consistent with the fact that the first has a putative binding site to the calcium-mediated conformation of a pectin structure [68], and the second is a promoter of pectin solubilization [69], and thus all these Prxs that have some level of relation with pectin are expected to be less numerous in type II walls. 

Associated with lignin biosynthesis, *At*Prx52 (AT5G05340) [70] and the monocot homologs (Os01g0205900, Bradi3g09120, SCQSST3114C09) were present in all analyzed cell wall proteomes. Similarly, the proteins related to lignification *At*Prx72 [71] (Os01g0327100, Bradi2g40590 and SCEPRZ1011A06) and *At*Prx64 [72] (Bradi2g37060 and SCJFLR1035D05) were identified in the monocots. *At*Prx16 (Os04g0656800, Bradi1g33740 and SCJFLR1035D02) and *At*Prx53 (Os10g0109300 and Bradi1g68900) were both identified among *A. thaliana* and the monocots’ CWPs. The first is related to germination [73] and the second to cell elongation inhibition and cell wall strengthening [74]. 

*At*Prx17 was also linked to lignin content when induced by the transcription factor AGAMOUS-LIKE15, which controls the lignification of tissues and changes the cell wall properties [75]. This could point to a conserved pathway in plants, as *At*Prx17 was identified in *A. thaliana* and in monocots´ CWP studies. Also identified in the four cell wall proteomes, *At*Prx39 was linked to higher production of reactive oxygen species that led to cold tolerance [76], evidencing the multiple roles of peroxidases in plant development. Because of the numerous functions of Prxs, more targeted studies are needed to determine the reason of their high variety in the plant cell wall, both in dicots and monocots.

BBEs catalyze the formation of berberine bridges, but in plants their function is vastly unexplored. In *A. thaliana*, it has previously been shown that some BBEs can be identified as monolignol oxidoreductases, and are related to lignin formation [77]. A much higher proportion of BBEs is found in *A. thaliana*. Phylogenetic analysis shows that not only does *A. thaliana* have more BBE members, but these enzymes also present several types of active sites, and few of type IV, which is exactly the one that is found most in grasses. In the course of evolution, in addition to expanding the amount of BBEs, it seems that the number of BBEs with active site type IV decreased and type I increased. Four BBEs only identified in *A. thaliana*—AT1G01980, AT4G20840, AT1G11770 and AT4G20830, the last with a type I active site—are able to inactivate oligogalacturonides, which is suggested to strengthen the immune response to fungal polygalacturonases [78], pointing to an evolved mechanism. Previously, it has been shown that BBEs in monocots seem to lack the catalytic and substrate coordination motifs linked to monolignol oxidoreductase activity, which was linked to lignin formation [77]. There are few functional studies in plant BBEs with type IV active sites, but fungal BBEs with this site have been related to oligosaccharide oxidation and plant immune response [79].

### 3.3. Proteins Related to Lipid Metabolism

Under this category, we highlight the Lipid Transfer Proteins (LTPs), Glycerophosphodiester Phosphodiesterases (GDPD)/GPDP-Like (GDPDL) and lipases GDSL. LTPs and lipases are proportionally more and less numerous in the monocot cell wall proteome, respectively [28,29,30,31,33,34,35,36,37,38,39,40,48]. LTPs are encoded by large multigenic families, which are considered to be essential to land colonization by plants, and are among the most abundant secreted proteins, but their exact in vivo role is still unclear. It has been suggested that LTPs mediate the transference and adhesion of molecules required for the composition of lipid barriers that are water-resistant, such as cutin, suberin and wax (reviewed by Edqvist et al. [80]). In the leaves of C4-metabolism plants, such as *Saccharum* spp. and *B. distachyon*, suberin surrounds the plasma membrane of bundle sheath cells, inhibiting CO_2_ diffusion [4], which could be a possible explanation for increased LTPs in these species. Accordingly, in *Saccharum* spp., some LTPs were only identified in leaves. LTPs were also associated with lipid deposition for cell expansion, as their transcripts were differentially expressed in maize elongating internodes in comparison to non-elongating ones [81]. Curiously, homologs of LTP12 were identified in *B. distachyon* (Bradi4g25750) and *O. sativa* (Os12g0115100), but not in *A. thaliana* (AT3G51590), where it was thought to be pollen-specific [82]. Monocot homologs of AT5G01870 were unique to the suspension cell culture or young plants, which could be an indication that it is related to growing tissues. In *B. distachyon* and *Saccharum* spp., AT5G01870 homologs seem to be organ-specific, and are found only in leaves. 

AtLTP3 was identified in all four cell wall proteomes analyzed. This protein negatively regulates plant defense mechanisms through the regulation of the antagonism between abscisic and salicylic acids, as it is induced by the former. LTP3 is proposed to be a disease-related marker, and it is also thought that LTP3 and 4 show some level of redundancy in plant immunity [83], but curiously, LTP4 was not identified in the monocots CWPs, and whether this overlay occurs in grasses could be an interesting research topic. Identified in all four species, the AtGDPDL3 (AT4G26690) is linked with lipid rafts in root-hair tip growth, suggesting that root hairs could be used as a model to study lipid rafts in plant development [84].

### 3.4. Proteases

Essentially, Ps break peptide bonds and control several relevant plant processes, such as protein transport, activity and half-lives [85], being generally divided into aspartyl (Asp), serine, cysteine, metallo and threonine proteases. The proportions of Asp Ps seem to be slightly higher in monocots. Phylogenetic analyses indicate that S8, C1A and A1 plant proteases functions were established even before the evolutive divergence of monocots and eudicots. This is corroborated by the conservation patterns of intron/exon arrangements and phylogeny analysis from monocots and dicots [85]. Accordingly, most of the proteases identified in the CWP studies from the monocots showed the highest identities with accessions also identified in the *A. thaliana* cell wall proteome (see Supplementary Tables S1–S4). However, only targeted functional analysis would reveal whether Ps display specific activities in monocots.

The senescence-associated subtilisin (AT3G14067) is a serine protease identified in all four species cell wall proteomes (Bradi2g51440/Bradi3g57140/Bradi3g57130, SCJFRT2057F03/SCRFHR1007E04, Os02g0779200), which confirms the fact that orthologs were identified in monocots crops [86]. Subtilisin was associated with the regulation of abscisic acid (ABA) signaling and drought tolerance [87], probably through a conserved mechanism between dicots and monocots, given the relevant role it plays.

The cysteine protease papain-like AtSAG2 (also named AALP) (AT5G60360) has been associated with senescence and necrotic cell death [88], as its expression increases along with leaf development. Reasonably, AtSAG2 was mostly identified in mature organs in *A. thaliana* and in the monocot cell wall proteomes, pointing to its use as a senescence marker in monocots. 

### 3.5. Proteins with Interacting Domains

IDs encompass Pectin Methyl Esterase Inhibitors (PMEIs), proteins with leucine-rich repeat (LRR) and Lysm domains, protease inhibitors such as cystatins, Bowman-Birk inhibitors, lectins and jacalins. PMEIs were proportionally less numerous in monocots, as might be expected for pectin-poor type II walls. Interestingly, Hocq et al. [89] showed that *At*PMEI9 is a strong inhibitor of *At*PME3, which were both identified in *A. thaliana* and *B. distachyon* cell wall proteomes. It has been suggested that PMEIs diverge with respect to their ability to bind PME at different pHs, which results in varied modulation of the pectin structure. Additionally, they act in pairs formed by the enzyme plus its inhibitor, an interaction mode more tightly controlled by the structural determinants from the inhibitor than the enzyme [89].

*O. sativa* showed a higher proportion of proteins with Lysm domains, which was associated with both plant immunity and symbiotic interactions in this species [90]. All Bowman-Birk serine protease inhibitors (BBIs) were only identified in members of Poaceae and Fabaceae families [91], and thus they have only been found in *Saccharum* spp. (SCJFLR1013A04 and SCRUFL3062D08), *B. distachyon* (Bradi2g24810, Bradi1g03510, Bradi2g01920 and Bradi2g24820) and *O. sativa* (Os01g0132000) cell wall proteomes. Protease inhibitors regulate protease activities, and BBIs, in particular, display an essential role in defense mechanisms directed towards protection against pathogens and pests [91]. Another type of protease inhibitor, *At*Cys6 homologs were identified in *B. distachyon* (Bradi2g52670) and *Saccharum* spp. (SCEPLR1051C09), which are associated with abiotic stresses and nucleic acid degradation [92].

Overall, the LRR-containing domain is conserved throughout evolution in the plants, displaying activity in the innate immune system through the sensing of pathogen-associated molecular patterns [93]. *At*PGIP1 (an LRR-domain protein) and its monocots corresponding accessions were identified in cell wall proteomes. *At*PGIP1 was associated with reduced damage caused by infection of a root nematode by inducing plant camalexin and indole-glucosinolate pathways [94]. The levels of the transcripts of another protein classified as ID, *At*Cys-5, are increased upon nematode infection [95] and ABA [96]. This cystatin was also identified in all four species’ CWP surveys. Perhaps both proteins could be part of the conserved defense mechanisms against nematodes in dicots and grasses.

### 3.6. Proteins Possibly Related to Signaling

This class of CWPs is composed by fasciclin-like arabinogalactans (FLAs), leucine-rich repeat receptor protein kinases (LRR-RKs) (actually trans-membrane proteins), and COBRA-like proteins (COBLs), among others. The proportion of proteins from the S class is similar in all four species, being higher in *B. distachyon* (see Figure 1). In proportion, FLAs seem to be more numerous in the cell wall proteomes of monocots. However, phylogenetic analyses showed that *A. thaliana* has 21 and *O. sativa* 15 FLA genes with conserved functions [97]. FLAs are related to cell-to-cell adhesion, mechanical strength for secondary cell walls and cellulose biosynthesis [98], in addition to elasticity [99]. Identified in all four species, FLA1 is supposed to act on the lateral root and shoot formation in tissue culture [100], and two *B. distachyon* FLAs (Bradi2g00220 and Bradi4g33490) were only identified in internodes, indicating that they could display organ-specific activities. In contrast, COBLs were only identified in *A. thaliana* CWPs, with the exception of one COBL in *O. sativa* (Os10g0497700). These are glycosylphosphatidylinositol-anchored specific plant proteins, and are associated with cell expansion and cellulose level of crystallinity, predominantly in elongating tissues [101]. More members of COBL are found in dicots, as several duplications occurred after the separation of dicots and monocots during evolution [102]. Recently, it has been demonstrated that a *Sorghum bicolor* COBL protein is linked to cellulose biosynthesis in the secondary wall, affecting plant mechanical strength [103], and this provided evidence of a cellulose-related role for this protein family in grasses. Overall, the LRR domain-containing proteins are conserved in both dicots and monocots [104].

### 3.7. Miscellaneous

Proteins with diverse functions are grouped under this category. Invariably, in several studies on CWPs, these consist of dirigent proteins, germins, thaumatins, gibberellic acid-stimulated proteins, purple acid phosphatases, phosphate-induced (phi) proteins, aldose epimerases, carbonic anhydrases, metallophosphoesterases, ribonucleases, pathogenesis-related proteins, low-molecular-weight cysteine-rich proteins and strictosidine synthases. Identified in all four species, *At*PAP10 has been proven to be transcriptionally regulated by MYB-CC factors, which control plant responses to inorganic phosphate starvation [105]. A germin protein (AT1G72610, and the corresponding Os08g0460000 and Bradi3g37680) seems to be leaf-specific, as it was mostly identified in this organ. Another protein from the same family (AT1G18970 and the corresponding Os03g0804500) was found to be auxin-responsive [106]. 

Dirigent proteins have more members in *B. distachyon* and *Saccharum* spp. CWP surveys. The family of dirigent proteins is possibly linked to lignin polymerization [107], and its higher content may be associated with the presence of aromatic molecules such as ferulic acids in some type II primary walls [4]. 

Nucleoside phosphatases were only identified in *Saccharum* spp. One of them, SCCCRZ1C01H06, is an apyrase associated with calcium signaling and has been suggested to be a messenger for sucrose accumulation [108], which is consistent with this species’ high sugar content. 

### 3.8. Structural Proteins

The reduced number of SPs in CWP studies on monocots is probably due their low levels in type II walls [6]. They are usually covalently linked to the wall, and thus present extra difficulty with respect to extraction. Extensins (EXTs), Proline- and Glycine-Rich Proteins are the most represented structural protein families in *A. thaliana* CWPs. In monocots, 10 leucine-rich repeat extensins (LRXs) have been identified altogether. EXTs are basic cell wall glycoproteins, rich in hydroxyproline residues with alternating hydrophilic and hydrophobic motifs [109], and have been associated with cell wall strengthening after different stresses and with pectin to create a coacervate that may serve as a template for cell wall deposition [110], reinforcing their role in type I cell expansion. Intriguingly, they are one of the most relevant families of CWPs in C3. In addition, specifically in *B. distachyon*, EXTs are essential to embryo regeneration and germination [111]. Three (Bradi2g05080, Bradi3g03370 and Bradi2g42477) of the four LRXs found in *B. distachyon* CWP studies [33,34,35,36] were also identified in the bioinformatic work of Liu et al. [112]. In a previous analysis, eight LRX genes were identified in *O. sativa* genome and were thought to form two distinct clades for vegetative and reproductive organs, which could reflect adaptations to different cell wall types. In addition to their role in cell expansion, it is suggested that LRXs might act on cell differentiation [113]. 

### 3.9. Proteins of Unkown Function

UFs mostly consist of several proteins with Domains of Unknown Function (DUFs). DUF642, for example, is present in the three monocot species plus *A. thaliana* (AT3G08030, Bradi1g04670, Os03g0807700, SCCCCL4009G04, AT5G11420, Os01g0611000, SCCCLB1001G04, AT5G25460 and AT4G32460). According to this categorization, it would only be speculative to propose for them a functional role, but this family is thought to be conserved, and has been proposed to be a new family of carbohydrate-binding proteins [114]. Some of these proteins have shown their ability to bind cellulose [114], have been shown to interact with PME [115], with auxin flux and hypocotyl elongation [116] and germination [117].

Several CWPs classified as UFs possess the BURP domain. Identified in *O. sativa* and *B. distachyon* (Os01g0733500 and Bradi2g49000), AT5G25610 has high sequence identity with *Gh*RDL1. *Gh*RDL1 interacts with a cotton α-expansin, and together they promote plant growth when overexpressed simultaneously [118]. The great number of UFs identified in the four species, and the fact that several UFs are suggested to have roles linked to the cell wall polysaccharides, reinforce the need for more functional studies on them to bring more information regarding the biology of monocot cell walls.

## 4. Applicative Aspects of Research on CWPs

Glycoside hydrolases are good candidates to be used in order to increase plant biomass or decrease recalcitrance destined to bioenergy production. Considering the enzymatic cocktails made from enzymes of microorganisms and the data on CWPs identified in monocots, the focus may be on GH families 1, 3, 17, 27, 35 and 51 [49]. The expression of a bacterial GH5 in *A. thaliana* led to a less recalcitrant wall without harming plant growth. It is suggested that the β-1,4 linkages of cellulose could be cut in the wall in an appropriate time and result in beneficial effects only [119]. As grasses present more GH5 and are the material used for biofuel production, they could be enzymes to watch with respect to their manipulation in the course of plant development. 

As Prxs might be involved with lignification, they need to be mentioned when discussing ways to improve biomass and to facilitate its conversion. In this sense, *OsPrx38* overexpression in *A. thaliana* increased biomass and seed yield under arsenic stress [120], which could be an alternative pathway to be engineered.

The overexpression of a plantacyanin (AT2G02850, an OR), identified in the cell wall proteomes of all species analyzed here, led to decreased plant biomass and seed yield in *A. thaliana*. As this gene is regulated through a microRNA (miR408) whose overexpression results in augmented biomass, this could be further studied [121]. 

In a previous work, Endo et al. [122] fused the promoter of an LTP gene (AT3G18280), identified in *A. thaliana* and monocot CWP studies, named *At*TED4, with several other genes. *At*TED4 was able to regulate the transition from the immature to the mature developmental stage, as expected for an early xylem-specific promoter, which could be used to target specialized genes in biomass engineering.

## 5. Conclusions

Although a comparison among a variety of studies, and modes of extraction, analysis and mass spectrometry techniques is still far from a complete understanding, this review compiled the most extensive studies on monocot CWPs, gathering their common characteristics and comparing them to *A. thaliana* data. As not all studies have used quantitative analysis, the abundance of these proteins should be considered in a more detailed way in the future before drawing conclusions. The different GH families and the low number of both pectin-related CWPs and SPs were related to the specific type II-wall characteristics, such as the presence of mixed β-d-glucans, and lower contents of pectin and structural proteins. Furthermore, specificities were indicated, such as the monocot proteins from the GH18 family, and some questions remain unanswered, such as the roles of the Prx, Bowman-Birk inhibitor and dirigent proteins in type II cell wall processes.

Despite the particularities displayed by type II cell walls, the nucleotide sequences of genes encoding some of the proteins that are lacking in grasses are conserved among dicots and monocots [123]. This observation points to the fact that grasses have the genetic capacity to produce xyloglucan, but it remains inactive [43]. Furthermore, we need to increase our knowledge of CWPs from both dicots and monocots to provide novel insights into the specialization of type II cell walls and their adaptive advantages. This would aid in genetically tailoring plants to improve efficiency and biomass for the production of commercially important products such as second-generation biofuels. In addition to the gains brought by the first surveys on the CWPs of monocots, data mining and integration using several -omics and protein–protein interaction studies are needed to establish the roles of CWPs.

## Figures and Tables

**Table 1:** Summary of the plant samples, extraction and mass spectrometry methods used in monocot CWP studies.

Species	Plant Sample	Extraction Method	Pipeline of MS Analyses	Total of CWPs Identified
*B. distachyon*	young and mature leaves, apical and basal internodes [33]	destructive technique followed by salt-based extraction	separation through 1D-PAGE and LC-MS/MS	594
	young seedlings [35]	destructive technique followed by SDS and phenol-based extraction, and enrichment by ConA affinity chromatography	2D-LC-MS/MS	
	seeds [36]	destructive technique followed by salt-based extraction	separation through 1D-PAGE and LC-MS/MS	
	seeds (9, 13 and 19 days after flowering (DAF)) [34]	destructive technique followed by salt-based extraction	separation through 1D-PAGE and LC-MS/MS	
*Saccharum* spp.	7 days cells suspension cultures [37]	destructive technique followed by salt-based extraction	2D-LC-MS/MS	283
	2 month-old internodes [38]	destructive and non-destructive techniques followed by salt-based extractions	2D-LC-MS/MS	
	4 month-old young and mature leaves and apical and basal internodes [39]	destructive technique followed by salt-based extraction	2D-LC-MS/MS	
	7 month-old young and mature leaves [40,48]	destructive and non-destructive techniques followed by salt-based extractions	2D-LC-MS/MS	
	7 month-old apical and basal internodes [40,48]	non-destructive technique followed by salt-based extraction	2D-LC-MS/MS	
*O. sativa*	5 days cultures medium of cell suspension cultures [28]	non-destructive technique followed by TCAAEB-based extraction	separation through 2D-PAGE and LC-MS/MS	270
	2–3 weeks cell suspension cultures [29]	non-destructive technique followed by salt-based extraction	separation through 1D-PAGE and 2D-LC-MS/MS	
	3 week-old 4th leaf [28]	non-destructive technique followed by Tween-20, CTAB and TCAAEB-based extraction	separation through 2D-PAGE and LC-MS/MS	
	2–3 weeks culture medium of cell suspension cultures [30]	non-destructive technique followed by salt-based extraction	separation through 1D-PAGE and 2D-LC-MS/MS	
	roots [31]	non-destructive technique followed by salt-based extraction	separation through 2D-PAGE and MALDI-TOF/TOF MS	

## Supplementary Materials

Supplementary materials can be found at https://www.mdpi.com/1422-0067/20/8/1975/s1. Table S1. List of CWPs identified in *A. thaliana* (retrieved from WALLPROTDB [48]) and the accessions from *B. distachyon*, *Saccharum* spp. and *O. sativa* identified through BLASTp analysis; Table S2. List of CWPs identified in *B. distachyon* (retrieved from WALLPROTDB [48]) and the *A. thaliana* accessions identified through BLASTp analysis; Table S3. List of CWPs identified in *Saccharum* spp. (retrieved from WALLPROTDB [48]) and the *A. thaliana* accessions identified through BLASTp analysis; Table S4. List of CWPs identified in *O. sativa* (retrieved from WALLPROTDB [48]) and the *A. thaliana* accessions identified through BLASTp analysis. The published and unpublished proteomics data were retrieved from WALLPROTDB [48]. Phytozome nomenclature [124] was used for *O. sativa*.

## Abbreviations

1D-PAGE	one-dimensional polyacrylamide gel electrophoresis
2D	two-dimensional
2D-PAGE	two-dimensional polyacrylamide gel electrophoresis
ABA	abscisic acid
Asp	aspartyl
BBE	berberine-bridge enzyme
BBI	bowman-birk serine protease inhibitor
CaCl2	calcium chloride
CWP	cell wall protein
COBL	COBRA-like protein
DAF	days after flowering
DUF	protein with domains of unknown function
ER	endoplasmic reticulum
EXT	extensin
FLA	fasciclin-like arabinogalactan
GDPD	glycerophosphodiester phosphodiesterase
GDPDL	glycerophosphodiester phosphodiesterase-like
GH	glycosyl hydrolase
ID	protein with predicted interaction domains
LiCl	lithium chloride
LC-MS/MS	liquid chromatography-tandem mass spectrometry
LM	protein related to lipid metabolism
LRR	leucine-rich repeat
LRR-RK	leucine-rich repeat receptor kinase
LRX	leucine-rich repeat extensin
LTP	lipid transfer protein
M	miscellaneous protein
MS	mass spectrometry
OR	oxidoreductase
P	protease
PAC	proteins acting on carbohydrate
PME	pectin methyl esterase
PMEI	pectin methyl esterase inhibitor
Prx	class III peroxidase
S	protein possibly involved in signaling
SP	structural protein
UF	proteins of unknown function
